# Antibiotic-induced gut microbiota dysbiosis in the PICU: mechanisms, clinical outcomes, and management strategies – a narrative review

**DOI:** 10.3389/fimmu.2026.1910105

**Published:** 2026-09-01

**Authors:** Cheng Yang, Dandan Wang, Wentao Peng

**Affiliations:** 1 Department of Paediatric Intensive Care Unit Nursing, West China Second University Hospital, Sichuan University, Chengdu, China; 2 Department of Paediatric Intensive Care Unit Nursing, WCSUH-Tianfu·Sichuan Provincial Children’s Hospital, Meishan, China; 3 Key Laboratory of Birth Defects and Related Diseases of Women and Children, Sichuan University, Ministry of Education, Chengdu, China; 4 The Second Affiliated Hospital of Chengdu Medical College, Nuclear Industry 416 Hospital, Chengdu, China; 5 Department of Nursing, West China Second University Hospital, Sichuan University, Chengdu, China; 6 Department of Nursing, WCSUH-Tianfu·Sichuan Provincial Children’s Hospital, Meishan, China

**Keywords:** antibiotic stewardship, dysbiosis, gut microbiota, microbiome, PICU, sepsis

## Abstract

**Background:**

Antibiotic exposure is highly prevalent in the paediatric intensive care unit (PICU) and constitutes a major modifiable driver of gut microbiota dysbiosis. However, a systematic synthesis focusing specifically on the PICU population has been lacking. This narrative review addresses how antibiotic exposure drives gut dysbiosis in the PICU, its clinical consequences with emphasis on immunological pathways, and its management.

**Methods:**

This review was informed by a structured search of PubMed, Web of Science, Cochrane Library, and Chinese databases up to June 2026, including studies on antibiotic exposure, microbiota alterations, clinical outcomes, and management in critically ill children.

**Results:**

Antibiotic use in PICU children ranges from 58% to 94%, with broad-spectrum and combination therapy being common. Anti-anaerobic antibiotics—particularly piperacillin-tazobactam, meropenem, and clindamycin—cause the most pronounced disruption, as quantified in adult ICU cohorts. In PICU children, clinical consequences include a higher incidence of Clostridioides difficile infection and an increased risk of ventilator-associated pneumonia following carbapenem exposure. Antibiotic stewardship, encompassing de-escalation and avoidance of unnecessary anaerobic coverage, is the first-line microbiota protection strategy. Probiotics reduce ventilator-associated pneumonia and shorten PICU stay, but should not be used routinely in high-risk children. High-fibre enteral nutrition has shown feasibility, whereas postbiotics and faecal microbiota transplantation lack PICU-specific trial data.

**Conclusions:**

Antibiotics are among the most significant modifiable drivers of gut dysbiosis in the PICU, with dose-dependent, class-specific effects. Antibiotic stewardship should be prioritised before any microbiota-directed intervention. Live probiotics require caution in high-risk populations, while non-live interventions are promising but need larger trials. Future research should employ longitudinal, multicentre studies with standardised reporting to elucidate host–microbe interactions in critically ill children.

## Introduction

Critically ill children in the paediatric intensive care unit (PICU) face multiple organ dysfunction, prolonged hospital stays, and various iatrogenic exposures. Their gut microbiota is consequently affected by underlying disease, systemic inflammation, intestinal ischaemia-reperfusion injury, and antibiotic use. In this review, dysbiosis is defined as a state of disrupted gut microbial ecology characterised by reduced diversity, loss of beneficial commensals, and overgrowth of potentially pathogenic organisms. Several factors unique to the PICU environment amplify the risk and consequences of antibiotic-induced dysbiosis beyond those observed in general paediatric wards or outpatient settings. Critical illness itself—characterised by systemic inflammation, impaired tissue perfusion, and altered mucosal immunity—compromises intestinal barrier function and modifies the gut microenvironment, rendering the microbiota more susceptible to external perturbations (1). Enteral nutrition practices, which are frequently interrupted or delayed in critically ill children, may further influence microbial composition and metabolite production (2). Additionally, PICU patients are commonly exposed to other microbiota-disrupting agents, including proton pump inhibitors, opioid analgesics, and broad-spectrum antiseptics, which may act synergistically with antibiotics to exacerbate ecological disruption. Prolonged hospitalisation in the high-acuity PICU environment also exposes children to nosocomial pathogens that can colonise the gut and expand under antibiotic selection pressure (3). These converging factors—critical illness, nutritional status, concomitant medications, and environmental exposure—collectively distinguish the PICU from other clinical settings and underscore the particular vulnerability of this population to antibiotic-associated gut dysbiosis.

The consequences of this dysbiosis extend beyond the gut lumen. The intestinal microbiota plays a critical role in the development and maintenance of the host immune system, including the differentiation of regulatory T cells (Tregs), the production of mucosal IgA, and the maintenance of systemic immune homeostasis (1). Disruption of this microbial–immune crosstalk can lead to local and systemic immune dysregulation, predisposing critically ill children to secondary infections, prolonged inflammation, and impaired recovery (1, 4). Understanding these immunological consequences is therefore essential for devising interventions that not only restore microbial ecology but also mitigate immune-mediated morbidity in PICU patients.

Epidemiological data confirm that antibiotic prescribing is extremely common in the PICU. A 10-centre point-prevalence study (2023, n = 1462) reported that 58% of PICU children received at least one antibiotic order (3). A multicentre US evaluation covering 55 ICUs found an overall antibiotic exposure rate of 82.1%, with 48% receiving empirical therapy (5). An Italian study (2023–2024) reported that 87.7% of children received at least one antibiotic (mean 1.6–1.8 agents per child) (6). A Swiss single-centre study (2024, n = 870) showed an overall antibiotic exposure of 59.8 DOT (days of therapy) per 100 patient-days, increasing from 55.2 in 2019 to 64.5 in 2021, with the highest exposure in infants aged 0–1 month (72.7 DOT/100 patient-days) (7). Despite this high prevalence, a systematic review specifically focusing on the PICU population remains lacking. Although a systematic review on antibiotic effects on the gut microbiota of preterm infants in the neonatal intensive care unit (NICU) has provided quantitative estimates (Mulinge et al., 2023: 21 studies, 878 preterm infants) (8), no comparable synthesis exists for the PICU.

Existing narrative reviews on the PICU microbiota have notable limitations. The review by Battaglini et al. (2025) centred on adults, with limited paediatric evidence (1). Fan et al. (2021) focused on enteral nutrition, mentioning antibiotics only in passing (2). The ARMORD study by Peto et al. (2025) evaluated more than 20 antimicrobials in adult ICU patients (9). Taylor et al. (2025) quantified the suppressive effect of anti-anaerobic antibiotics on butyrate-producing bacteria (4.01% decrease per 48 hours) in 110 mechanically ventilated adult ICU patients (4). However, quantitative relationships between antibiotic type, dose, and duration and gut microbiota disruption specifically in the PICU population, as well as their impact on clinical outcomes, remain poorly integrated. Children differ from adults in their developing microbiota, immune system, and underlying diseases, necessitating a PICU-focused synthesis.

This narrative review addresses three core questions: how antibiotic exposure drives gut microbiota dysbiosis in the PICU, what the clinical consequences are, and how this condition should be managed. To inform this review, we conducted a structured literature search of PubMed, Web of Science, Cochrane Library, and Chinese databases (CNKI, Wanfang Data, and CQVIP) from inception to June 2026.

## Methods

### Study design

This article is a narrative review that synthesizes and interprets the current evidence on antibiotic-induced gut microbiota dysbiosis in the paediatric intensive care unit (PICU). Given the heterogeneity of the available literature, which includes observational studies, randomized controlled trials, systematic reviews, and clinical guidelines, a narrative approach was chosen to provide a broad, clinically oriented overview of the topic.

### Literature search strategy

We performed a structured literature search of the following electronic databases from their inception to June 2026: PubMed, Web of Science, Cochrane Library, and Chinese databases (CNKI, Wanfang Data, and CQVIP). The search strategy combined keywords and Medical Subject Headings (MeSH) terms related to three domains:

- Antibiotic exposure: (“antibiotic” OR “antimicrobial” OR “anti-bacterial agents”[MeSH]) AND (“exposure” OR “use” OR “therapy” OR “stewardship”)
- Gut microbiota: (“gut microbiota” OR “gut microbiome” OR “intestinal microbiota” OR “dysbiosis” OR “gastrointestinal microbiome”[MeSH])
- Population: (“paediatric intensive care unit” OR “PICU” OR “critically ill children” OR “children” OR “infant” OR “adolescent”)

The search was limited to human studies published in English or Chinese. Reference lists of included articles and relevant reviews were manually screened to identify additional studies.

### Inclusion and selection criteria

We included studies that reported on: (1) antibiotic exposure patterns in the PICU; (2) gut microbiota alterations (diversity, composition, or functional changes) associated with antibiotic use in critically ill children; (3) clinical outcomes linked to antibiotic-induced dysbiosis in paediatric populations; and (4) management strategies, including antibiotic stewardship and microbiota-targeted interventions (probiotics, prebiotics, postbiotics, faecal microbiota transplantation). We prioritized quantitative studies, systematic reviews, meta-analyses, and major clinical guidelines. We excluded animal studies, *in vitro* experiments, conference abstracts, and case reports. Studies focusing exclusively on adult populations were included only when paediatric data were unavailable, and were explicitly identified as adult-derived evidence.

### Data synthesis

Because this is a narrative review, we did not perform a formal quality assessment, meta-analysis, or quantitative synthesis. Instead, we extracted key findings from the included literature, organised them around the three core questions, and contextualized them within PICU clinical practice.

### Extrapolating adult evidence to PICU children: key considerations

Throughout this review, we draw on evidence from adult ICU and neonatal studies where PICU-specific data are limited. However, several differences between these populations warrant caution when extrapolating findings to PICU children.

First, the infant gut microbiota differs fundamentally from that of adults. Mulinge et al. (8) described the neonatal gut microbiota as characterized by low diversity and high temporal instability during early life, making it potentially more vulnerable to external perturbations. In contrast, the adult gut microbiota is relatively stable, as summarized by Battaglini and Torres (1), suggesting that findings from adult cohorts may not fully reflect the ecological vulnerability of the developing paediatric gut.

Second, critically ill children admitted to the PICU differ from adults in their underlying conditions and illness trajectories. As discussed in the Introduction, children face distinct developmental, immunological, and disease-specific factors that may modify their response to both antibiotic exposure and microbiota disruption.

Third, pharmacokinetic handling of antibiotics differs substantially across age groups. Fenner et al. (7) reported that the highest antibiotic exposure in their Swiss PICU cohort occurred in infants aged 0–1 months (72.7 DOT/100 patient-days), reflecting age-dependent variations in drug metabolism and clearance that are not present in adult populations.

Despite these important differences, adult studies provide valuable directional hypotheses and quantitative frameworks—particularly regarding dose–response relationships between specific antibiotic classes and microbiota disruption—that can guide PICU research and practice where paediatric data are sparse. We have explicitly identified adult-derived and neonatal-derived evidence throughout the manuscript and urge readers to interpret these findings with appropriate caution.

### Age-dependent susceptibility to antibiotic-induced dysbiosis in PICU children

While the preceding section addressed the extrapolation of adult data to children, it is equally important to recognize that the PICU population spans a broad developmental spectrum, and susceptibility to antibiotic-induced gut dysbiosis likely varies substantially by age.

Infants (<1 year) appear to be at the highest risk. The neonatal and infant gut microbiota is characterized by low diversity and high temporal instability, making it inherently more susceptible to external perturbations (8). This developmental vulnerability is compounded by the highest antibiotic exposure rates observed in this age group: Fenner et al. (7) reported that infants aged 0–1 months had the highest antibiotic exposure in their Swiss PICU cohort (72.7 DOT/100 patient-days), substantially exceeding older children. In very preterm infants, antibiotic use >7 days within the first 28 days of life was associated with more severe dysbiosis and slower recovery of diversity (10), whereas shorter courses (≤2 days) did not disrupt microbiota development (11), suggesting a possible “safe window” in neonates. Whether similar windows exist for older PICU children remains unknown.

For toddlers (1–3 years) and older children and adolescents (>3 years), direct evidence is extremely limited. The adult gut microbiota is relatively stable (1), and by inference the microbiota of older children may be more resilient than that of infants, but age-stratified PICU data are not available to confirm this. The available evidence from older children—such as a high-fibre enteral nutrition pilot study with a mean age of 10.8 years (12)—provides only indirect insights and did not perform age-stratified analyses.

A critical evidence gap exists across all paediatric age groups: most PICU studies to date have aggregated children from infancy to adolescence without age-stratified analyses (3, 5, 13, 14), precluding direct comparisons of differential susceptibility. If infants are indeed more vulnerable, they may warrant more stringent antibiotic stewardship and closer monitoring of gut function, whereas older children may tolerate similar exposures with less disruption—though this remains speculative without confirmatory studies. Future research must explicitly perform age-stratified analyses to define the differential susceptibility across paediatric age groups and guide age-appropriate management strategies.

### Antibiotic exposure and gut microbiota dysbiosis in the PICU

#### Patterns of antibiotic use in the PICU

Antibiotic use in PICU children ranges from 58% to 94%, with empirical, broad-spectrum, and combination therapy being common (3, 5–7). A retrospective multicentre study by Herawati et al. (2026) found that antimicrobial use density in the PICU (37.56 DDD (defined daily doses)/100 bed-days) was approximately seven times that in the same institution’s NICU (5.22 DDD/100 bed-days). In the private university hospital’s PICU, “Watch” group broad-spectrum antibiotics (mainly ceftriaxone and meropenem) accounted for 71.27% of prescriptions. Using generalized linear modelling, the same study showed that rational antibiotic therapy was significantly associated with a 17% reduction in length of hospital stay (β = -0.187, p = 0.002) (15).

Inappropriate antibiotic use is also concerning. A prospective Indian study (2025) reported that 41.4% of children received only a single antibiotic (mean 1.8 per child), with third-generation cephalosporins being the most frequently prescribed (50.7%), which contributes to antimicrobial resistance, adverse reactions, increased morbidity, and higher costs (16).

#### Impact of antibiotic class on microbiota disruption

The ARMORD study (Peto et al., 2025) systematically evaluated more than 20 antimicrobials. Recent use of piperacillin-tazobactam, meropenem, intravenous co-amoxiclav, and clindamycin was associated with the greatest reductions in microbial diversity and anaerobic abundance. Exposure to piperacillin-tazobactam and meropenem was also linked to increased enterococcal abundance and expansion of resistance genes. Notably, some WHO “Access” group agents (e.g., co-amoxiclav, clindamycin) had an impact comparable to “Watch” group drugs, challenging the simplistic use of the AWaRe classification alone (9).

Taylor et al. (2025) provided quantitative evidence from an adult ICU cohort: each additional 48 hours of exposure to anti-anaerobic antibiotics (piperacillin-tazobactam, co-amoxiclav, metronidazole, meropenem) was associated with a 4.01% decline in butyrate-producing bacteria (95% CI: 2.25%–5.84%; p < 0.0001) and a 6.98% increase in pathobionts—commensal microorganisms that have the potential to cause disease under certain conditions, such as immunosuppression or loss of colonization resistance (95% CI: 4.07%–9.89%; p < 0.0001). The odds of >30% expansion of any single pathobiont increased 2.32-fold (95% CI: 1.35–4.01; p = 0.0026) (4). Clinically, these findings suggest that every two days of anti-anaerobic antibiotic therapy incrementally compromises the gut’s capacity to produce butyrate—a key metabolite for maintaining intestinal epithelial integrity and regulating mucosal inflammation—while simultaneously promoting the expansion of potentially pathogenic organisms. This dose-dependent ecological shift provides a mechanistic rationale for limiting the duration of anaerobic antibiotic coverage whenever clinically feasible.

A prospective study by Xu et al. (2023) using metagenomic next-generation sequencing (mNGS) in PICU children receiving broad-spectrum antibiotics showed that after seven days, gut microbiota richness decreased significantly (p < 0.05), with alterations in multiple metabolic pathways and widespread upregulation of antimicrobial resistance genes, including macrolide (Erm(A), ErmX), tetracycline (tetO), and multidrug efflux (adeJ, adeF) resistance determinants (14).

#### Antibiotic duration and microbiota disruption

A systematic review and meta-analysis by McDonnell et al. (2021) (17 studies) confirmed that antibiotic exposure is the strongest correlate of gut dysbiosis in children, with a dose-dependent effect (17).

In a cohort of critically ill adults, Burrows et al. (2025) (126 adults, 71.4% meeting sepsis criteria) quantified the relationship: each additional day of antibiotic therapy was associated with approximately 0.93 more hospital days (p < 0.001). Clinically, this near-one-day increment per antibiotic day is cumulative: a 7-day course translates to approximately 6.5 additional hospital days at the population level, underscoring that even seemingly modest per-day effects carry substantial cumulative consequences for bed utilization and healthcare costs. Longer duration did not reduce one-year mortality or readmission rates (18), challenging the conventional belief that “prolonging the course guarantees safety.”

In very preterm infants, a 2024 study showed that antibiotic use >7 days within the first 28 days of life was associated with more severe dysbiosis and slower recovery of diversity (10). Aoki et al. (2026) found that short (≤2 days) antibiotic exposure in neonates did not disrupt microbiota development, whereas longer exposure (≥3 days) was associated with transient dysbiosis (11), suggesting a “safe window” in neonates. Whether a similar window exists in the PICU remains to be investigated.

#### Core mechanisms and quantitative evidence

**Figure 1:**
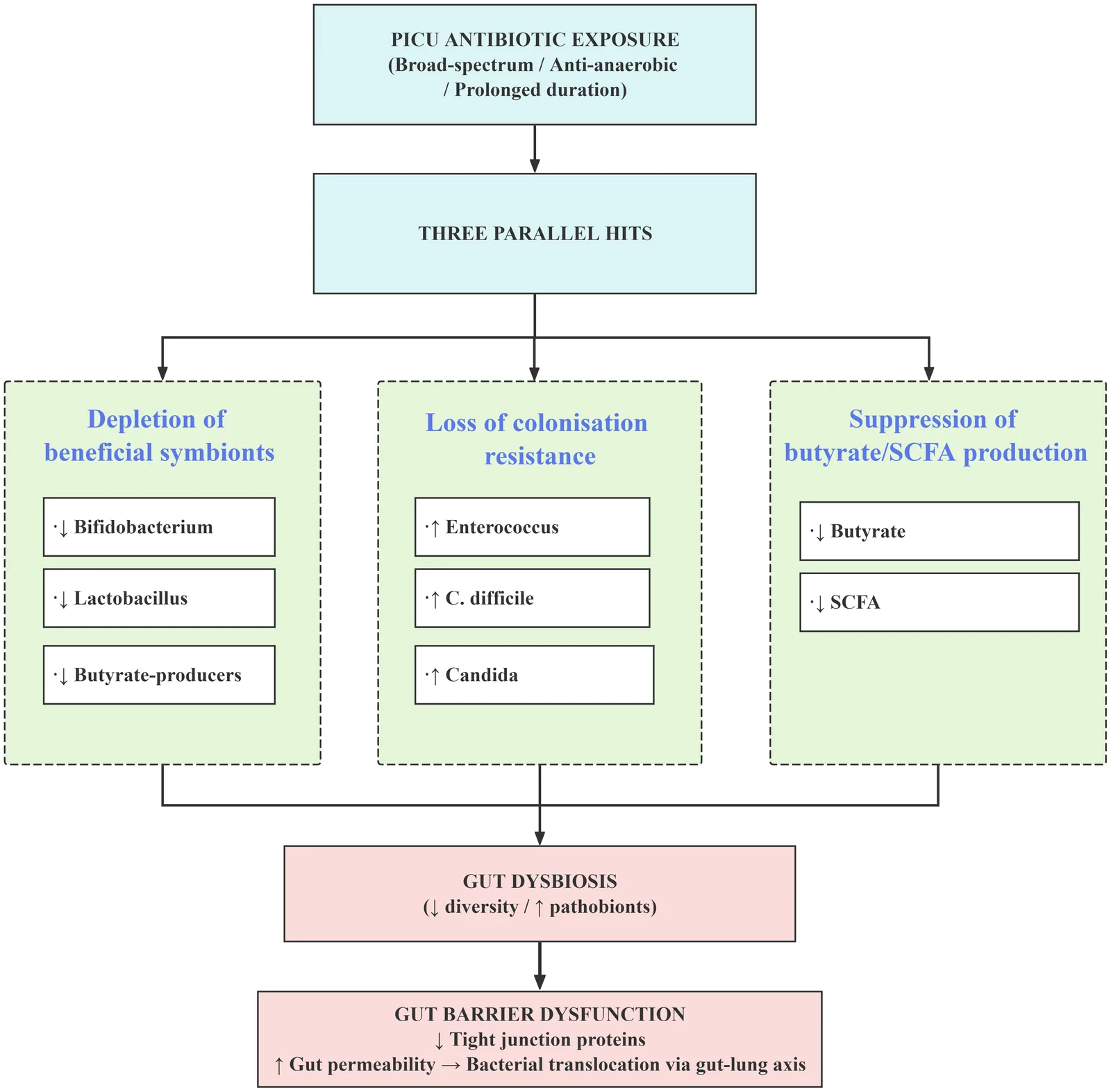
Mechanistic pathways of antibiotic-induced gut microbiota dysbiosis in the paediatric intensive care unit (PICU). PICU antibiotic exposure triggers three parallel hits: (1) depletion of beneficial symbionts (Bifidobacterium, Lactobacillus, butyrate-producers); (2) loss of colonisation resistance with pathobiont expansion (Enterococcus, C. difficile, Candida); (3) suppression of butyrate/SCFA production. These converge into gut dysbiosis and gut barrier dysfunction, leading to bacterial translocation via the gut-lung axis.

Antibiotics disrupt the intestinal ecosystem through several interconnected mechanisms. Sustained systemic antibiotic exposure in critically ill children may affect the recovery rate of gut microbial activity (6). The main mechanisms include:

1. Elimination of beneficial symbionts: Loss of Bifidobacterium, Lactobacillus, and butyrate-producing Firmicutes reduces microbial diversity and alters community structure.
2. Breakdown of ecological barriers: When commensals are depleted, opportunistic pathogens (enterococci, Candida, Clostridioides difficile) expand. McDonnell et al. (2021) confirmed that antibiotic exposure reduces commensal genera (Bifidobacterium, Lactobacillus) and increases potential pathogens (Enterococcus, Escherichia) in the paediatric gut (17).
3. Metabolic reprogramming toward inflammation: Taylor et al. (2025) quantified this effect in an adult ICU cohort: each additional 48 hours of anti-anaerobic antibiotics reduced butyrate producers by 4.01% (4). Decreased SCFA levels downregulate tight junction proteins and increase gut permeability, creating a mechanistic link between antibiotic exposure and the increased risk of bacterial translocation and secondary infections observed clinically.
4. Amplification of injury over time: McDonnell et al. (2021) showed a clear dose-effect relationship between antibiotic exposure and reduced diversity in children, with risk increasing for each additional antibiotic class used (17). Burrows et al. (2025) provided quantitative adult data (18).
5. Synergistic interaction with critical illness: Gut dysbiosis in critically ill patients is characterized by reduced diversity, loss of beneficial bacteria, and overgrowth of pathogens, leading to long-term immunosuppression and increased infection risk (1).
6. Immune dysregulation and impaired host defence: Antibiotic-induced suppression of butyrate-producing bacteria disrupts the production of short-chain fatty acids (SCFAs), which are critical for immune homeostasis. In early life, SCFAs promote the differentiation of regulatory T cells (Tregs) and support mucosal IgA responses, both essential for maintaining tolerance to commensals and defence against pathogens. Loss of this immunoregulatory network impairs local barrier immunity and may predispose to systemic inflammation and secondary infections (19). The interplay among these pathways is illustrated in **Figure 1**.

### Clinical consequences of antibiotic-induced dysbiosis

#### Clostridioides difficile infection

A global systematic review and meta-analysis (2016–2024, 59 studies, 24 countries) reported an incidence of hospital-onset, healthcare-associated CDI of 5.31 per 1000 admissions (95% CI 3.76–7.12) and 5.00 per 10,000 patient-days. Notably, the paediatric risk was 4.52 per 1000 admissions, higher than in adults (2.13 per 1000) (20).

A multicentre Canadian study by Baldassarre et al. (2024) (286 paediatric patients) found that recurrent CDI (rCDI) incidence was 12.9%, with an rCDI rate of 2.6 per 1000 at-risk days among hospitalized patients. Immunocompromised patients had a higher rCDI rate (17.5%; p = 0.03) and an increased rCDI risk independent of initial antibiotic therapy (OR 2.31; 95% CI 1.12–5.09) (21). Recent antibiotic exposure and hospitalization remain key risk factors for CDI in children (3, 21). US CDC NHSN data (2020–2022) showed a paediatric standardized infection ratio (SIR) for CDI of 0.88 in 2021 (22). The Chinese CDI guideline (2024) recommends stopping antimicrobials suspected of triggering CDI while receiving anti-CDI therapy (strong recommendation, moderate-quality evidence) (23).

#### Gut barrier dysfunction and bacterial translocation

SCFAs, particularly butyrate, upregulate intestinal epithelial tight junction proteins and decrease gut permeability. Taylor et al. (2025) showed that each additional 48 hours of anti-anaerobic antibiotics reduced butyrate producers by 4.01%, directly linking to decreased SCFA levels (4).

An open-label pilot study by O’Connor et al. (2024) (n = 20 children with septic critical illness, mean age 10.8 years) demonstrated that a high-fibre, food-based enteral formula maintained faecal butyrate and propionate concentrations and reduced stool frequency from 2.6 to 1.2 per day after one week (p < 0.004); 25% of children received more than 2 antibiotics during their ICU stay. This was the first UK study to report faecal SCFA concentrations in critically ill children receiving a food-based formula, providing preliminary evidence that nutritional support may protect the gut barrier (12).

Beyond physical barrier disruption, butyrate deficiency has been linked to impaired immune function, including compromised differentiation of regulatory T cells (Tregs) and reduced mucosal IgA production, both essential for maintaining immune tolerance to commensal microbiota and for effective defence against enteric pathogens (1). In the PICU setting, where children are exposed to antibiotics during critical windows of immune development, the potential for sustained immunomodulatory effects warrants urgent investigation.

#### Secondary infections and ventilator-associated pneumonia

Dysbiosis transforms the gut into a reservoir of opportunistic pathogens, which can spread via the gut-lung axis. A survey of VAP in a children’s hospital PICU (2018–2022) reported a VAP incidence of 10.3 per 1000 ventilator-days. Children with VAP had significantly longer PICU stays (25.0 vs. 10.0 days, p < 0.001) and longer mechanical ventilation (15.0 vs. 5.0 days, p < 0.001). Prior carbapenem exposure was an independent risk factor (OR 3.2; 95% CI 1.1–9.6), with Gram-negative bacteria being predominant (13). Clinically, this three-fold increased odds means that for every 10 children receiving carbapenems, approximately 1 additional case of VAP could be attributed to the antibiotic exposure itself—highlighting that even “appropriate” carbapenem use carries a quantifiable risk of subsequent pulmonary infection. Taylor et al. (2025) further showed a 6.98% increase in pathobiont abundance and a 2.32-fold higher odds of >30% expansion of any single pathobiont (p = 0.0026) (4).

A systematic review and meta-analysis by Wang et al. (2025) (12 RCTs, n = 1051 critically ill children) reported that probiotic use reduced VAP/HAP incidence (11.9% vs. 25.4%; RR 0.48, 95% CI 0.30–0.77, p = 0.002; low-certainty evidence), shortened PICU length of stay by approximately 2 days (moderate-certainty evidence), and reduced total hospital stay by approximately 6 days (low-certainty evidence). Clinically, these estimates suggest that for every 100 critically ill children treated with probiotics, approximately 13 fewer cases of VAP would occur, and the average child would be discharged from the PICU nearly 2 days earlier. However, the low-to-moderate certainty of this evidence, coupled with the documented safety risks in vulnerable subgroups—particularly children with septic shock, severe immunodeficiency, short bowel syndrome, or central venous catheters—argues against routine use and underscores the need for larger, confirmatory trials. The meta-analysis explicitly stated that the benefits need confirmation in larger RCTs (24).

#### Prolonged PICU length of stay

Burrows et al. (2025) confirmed that each additional antibiotic day was associated with approximately 0.93 more hospital days in critically ill adults (18). Herawati et al. (2026) showed that rational antibiotic therapy was associated with a 17% shorter length of stay (β = -0.187, p = 0.002) (15). The Swiss PICU study also reported higher antibiotic exposure in children with longer stays (65.1 DOT/100 patient-days for stays >2–7 days vs. 59.0 for stays <2 days) (7). A 2023 study from an Indonesian PICU demonstrated that clinical pharmacist participation in prescription review significantly reduced both PICU costs and length of stay (25).

#### Health economics of antibiotic exposure

A micro-costing analysis of a nationwide Brazilian infection prevention project (BMJ Open, 2025) showed that implementing standardized prevention measures in paediatric and neonatal ICUs was cost-saving (26). A 2025 study by Juang et al., with Kollef as a co-author, also noted that inappropriate antibiotic therapy is associated with increased mortality, prolonged hospital stay, and increased cost (27). These findings highlight the economic implications of antibiotic stewardship in the PICU. The key clinical outcomes associated with antibiotic-induced dysbiosis, along with their underlying mechanisms and supporting evidence, are summarised in **Table 1**.

**Table 1:** Clinical outcomes associated with antibiotic-induced dysbiosis in the PICU.

Clinical outcome	Core mechanism	Key evidence	Evidence base
*Clostridioides difficile* infection	Loss of colonisation resistance	Paediatric CDI incidence 4.52/1000 admissions, higher than adults (2.13/1000) (20)	PICU−specific (global meta−analysis with paediatric subgroup; direct evidence)
Ventilator-associated pneumonia (VAP)	Gut-lung axis; pathogen translocation	PICU VAP incidence 10.3/1000 ventilator-days. Prior carbapenem exposure: OR 3.2 (95% CI 1.1–9.6) (13)	PICU−specific (single−centre study; needs multicentre validation)
Prolonged PICU stay	Cumulative effect of antibiotic days	Each additional antibiotic day: +0.93 hospital days (P<0.001) (18). Rational antibiotic therapy: 17% shorter stay (β= –0.187, P = 0.002) (15)	Mixed (dose−response from adult data; stewardship benefit from PICU study)
Antibiotic-associated diarrhoea	Reduced diversity, pathogen overgrowth	Control group diarrhoea rate 19.6% in paediatric probiotic RCTs (36)	PICU−specific (paediatric RCTs; limited to control−group data)

### Clinical management strategies

#### A framework for approaching antibiotic-induced dysbiosis in the PICU

The management of antibiotic-induced gut dysbiosis in the PICU requires a hierarchical, risk-stratified approach that recognizes both the primacy of antibiotic stewardship and the evolving evidence for microbiota-targeted therapies. At the top of this hierarchy is the optimization of antibiotic use—reducing unnecessary exposure, shortening durations where safe, and avoiding agents with the greatest ecological impact. This first step is supported by the strongest evidence and carries no direct patient risk. Below this, microbiota-targeted interventions should be considered only after antibiotic use has been optimized, and their application must be guided by patient risk status. In practical terms, we propose that clinicians consider five questions when evaluating any microbiota-directed intervention for a PICU child: (1) Has the antibiotic regimen been optimized? (2) Is the child in a high-risk category that precludes live probiotics? (3) What is the strength of evidence for the proposed intervention in the specific PICU population? (4) Does the child have any contraindications to the intervention? (5) If live organisms are contraindicated, could a non-live alternative (e.g., high-fibre nutrition) be considered? This framework is intended to provide a structured, clinically actionable approach to a complex and rapidly evolving field.

##### Antibiotic stewardship as the first line of microbiota protection

Core principle: before any microbiota-directed intervention, optimizing antibiotic use should be the first step.

1. Shorten antibiotic duration. Burrows et al. (2025) showed that prolonging therapy does not reduce mortality but extends hospital stay (18). A 2026 systematic review (9 RCTs, 2329 adult patients) found no significant difference in mortality (OR 0.74), clinical cure, or microbiological clearance between short-course (7–10 days) and long-course (≥14 days) therapy for Gram-negative bloodstream infection (28). A 2023 meta-analysis on VAP in adults demonstrated non-inferiority for mortality, though short courses might increase recurrence of non-fermenting Gram-negative VAP (29). In adults with intra-abdominal infection who had adequate source control and negative blood cultures, a fixed 4-day course was non-inferior to longer courses (30).
2. Avoid unnecessary anaerobic coverage. Piperacillin-tazobactam, clindamycin, and carbapenems should be used with caution unless clearly indicated. The ARMORD study confirmed these drugs are associated with the greatest microbiota disruption (9), and Taylor et al. quantified their suppression of butyrate producers (4).
3. De-escalation. Once culture and susceptibility results are available, narrow the spectrum and stop unnecessary combination therapy. An Italian PICU intervention (2025) reduced overall antibiotic consumption by 3.0% per month (p < 0.0001), with meropenem down 4.9% (p = 0.009), glycopeptides 3.8% (p = 0.014), and piperacillin-tazobactam 4.8% (p = 0.034) (31). Clinically, this translates to a reduction of approximately one antibiotic day for every 33 patient-days, demonstrating that targeted ASP interventions can achieve meaningful reductions in broad-spectrum antibiotic exposure without compromising patient care.
4. Biomarker-guided discontinuation. The PROGRESS trial (2,660 critically ill adults) showed that procalcitonin-guided discontinuation significantly reduced antibiotic exposure without increasing 28-day mortality (32). A 2025 systematic review on neonatal sepsis found that shorter, biomarker-guided courses are effective without increasing relapse rates (33).
5. Implement antimicrobial stewardship programs (ASPs). Liberati et al. (2025) noted that PICU ASP implementation remains limited, with heterogeneous interventions and metrics, but preliminary positive effects have been observed (31). Pharmacist involvement significantly reduced PICU costs and length of stay (34).

Despite the theoretical benefits of antibiotic stewardship, its implementation in the PICU faces several practical challenges. First, diagnostic uncertainty is pervasive in critically ill children, where clinical signs of infection and non-infectious systemic inflammation frequently overlap (3). This drives clinicians toward broad empirical coverage, particularly when rapid diagnostic results are unavailable. Second, resource limitations—including limited access to rapid molecular diagnostics, insufficient microbiology laboratory capacity, and the absence of dedicated antimicrobial stewardship teams in many PICUs—hinder timely de-escalation and discontinuation (31). Third, there is often resistance to de-escalation among clinicians, rooted in a “better safe than sorry” culture and fear of clinical deterioration, especially when the consequences of undertreatment are perceived as more immediate than those of overtreatment. Finally, the paucity of PICU-specific data on optimal antibiotic durations for common infections leaves clinicians without clear, evidence-based stopping rules, forcing reliance on adult-derived guidelines that may not fully apply to children (9, 28–30). Acknowledging these barriers is essential for designing feasible and effective stewardship interventions tailored to the PICU environment.

##### Current evidence on microbiota-targeted interventions

1. Probiotics. Live probiotics should be used with extreme caution in PICU children and are absolutely contraindicated in those with septic shock, severe immunodeficiency, short bowel syndrome, or central venous catheterization. A 2025 meta-analysis reported 8 cases of probiotic-related sepsis among more than 20,000 exposed infants (incidence <0.04%), underscoring that even rare adverse events are clinically significant in vulnerable populations (35). A critical caveat when interpreting probiotic evidence is that different strains and species—such as Lactobacillus rhamnosus GG, Saccharomyces boulardii, Bifidobacterium spp., and multi-strain formulations—may have distinct biological effects, safety profiles, and clinical efficacy. However, most PICU probiotic trials have aggregated results across different strains without reporting strain-stratified outcomes, precluding direct comparisons. Therefore, the estimates presented here (RR 0.48 for VAP reduction; 1.94-day reduction in PICU stay) represent an average effect across heterogeneous formulations and should not be interpreted as evidence that all probiotic products are equally effective or safe. Until strain-specific RCTs are available, clinicians should exercise particular caution when selecting probiotic products, as formulations vary widely in composition, viability, and quality between manufacturers. In carefully selected PICU children without these contraindications, a meta-analysis of 12 RCTs (n = 1051) reported reduced VAP/HAP incidence (RR 0.48, 95% CI 0.30–0.77; low-certainty evidence) and shortened PICU stay by 1.94 days (moderate-certainty evidence), but found no effect on mortality (moderate-certainty evidence) (36). However, the low-to-moderate certainty of this evidence, coupled with the documented safety risks in high-risk subgroups, argues against routine use. Benefits, if any, are likely confined to select populations without contraindications, and confirmation in larger, adequately powered RCTs is urgently needed.
2. Prebiotics/high-fibre enteral nutrition. O’Connor et al. (2024) pilot study (n = 20) showed that a high-fibre, food-based enteral formula maintained faecal butyrate and propionate concentrations and reduced stool frequency from 2.6 to 1.2 per day (p < 0.004). This was the first UK study reporting faecal SCFA concentrations in critically ill children receiving a food-based formula, but the findings require confirmation in larger RCTs (12).
3. Postbiotics and faecal microbiota transplantation (FMT). Postbiotics (direct supplementation of SCFAs) can restore barrier function without live organism risk, but human PICU trials are lacking. FMT is effective for recurrent CDI in children, but no clinical trials exist for PICU children with sepsis, and safety risks (pathogen transmission, aspiration) remain (13, 36). The evidence base and safety considerations for each microbiota-targeted intervention are summarised in **Table 2**.

**Table 2:** Microbiota-targeted interventions in the PICU: evidence and safety.

Intervention	Core mechanism	Evidence (level)	Safety / precautions
Antibiotic stewardship	Reduce selective pressure	High	First-line strategy; prioritise before any other intervention
Probiotics (live)	Restore colonisation resistance	Low to moderate	Safety first: absolute contraindications — septic shock, severe immunodeficiency, short bowel syndrome, central venous catheterisation. Even in eligible children, benefits are supported by low−to−moderate certainty evidence and do not support routine use.
High-fibre enteral nutrition	Fermentable fibre → SCFA	Very low	Safe; monitor for abdominal distension; not for ileus
Postbiotics	Direct SCFA supplementation	None (PICU)	No clinical trials in PICU; not for routine use
Faecal microbiota transplantation (FMT)	Complete microbiota restoration	Limited to rCDI	Research only; risks: pathogen transmission, aspiration, bacteraemia

Evidence levels are classified as follows: High — consistent findings from multiple RCTs or high−quality systematic reviews; Low to moderate — limited RCTs with methodological concerns or inconsistent findings; Very low — pilot studies, single−centre observational data, or animal models only; None (PICU) — no PICU−specific data available; Limited to rCDI — evidence only for recurrent Clostridioides difficile infection, not for sepsis or other PICU indications. High−risk PICU children should not receive live probiotics routinely.

## Conclusions

Antibiotics are among the most significant modifiable drivers of gut microbiota dysbiosis in the PICU, with clear class-specific, dose-dependent effects supported by evidence from both PICU cohorts and, where paediatric data are limited, adult ICU studies. However, direct comparative data with other modifiable factors—such as enteral nutrition, proton pump inhibitors, or opioid analgesics—are lacking, and the relative contribution of antibiotics versus these other exposures remains to be quantified in future studies. Antibiotic stewardship—encompassing de-escalation, shortening of treatment duration where safe, and avoidance of unnecessary anaerobic coverage—should be prioritized as the first line of microbiota protection before any microbiota-directed intervention. Live probiotics should not be used routinely in high-risk children (those with septic shock, severe immunodeficiency, short bowel syndrome, or central venous catheterization) because the benefits are supported by low-certainty evidence and require confirmation in larger RCTs. Non-live interventions such as high-fibre enteral nutrition and postbiotics are promising but lack PICU-specific trial data. Future research must adopt large, multicentre prospective cohorts, multi-omics integration, and personalized strategies to translate these concepts into bedside practice. Studies evaluating the feasibility and effectiveness of stewardship interventions in diverse PICU settings are also urgently needed, particularly those addressing the practical barriers identified above.

Final message for clinicians:

Before considering any microbiota-directed therapy for a PICU child, ask:

- Has antibiotic therapy been optimized (de-escalated, shortened, anaerobic coverage avoided)?
- Is the child in a high-risk category (septic shock, severe immunodeficiency, short bowel syndrome, central venous catheterization)? If yes, live probiotics are absolutely contraindicated. If uncertain, seek infectious disease or gastroenterology consultation before considering probiotic use.
- Could a non-live intervention (high-fibre enteral nutrition, postbiotics) be considered instead?

## Key limitations and future directions

Current research on antibiotic-induced gut dysbiosis in the PICU has several key limitations, summarized in **Table 3**. These can be grouped into five overarching themes: evidence source and generalizability, confounding and comparative data, intervention trial methodology, population heterogeneity, and outcome assessment.

**Table 3:** Key limitations and future directions in PICU antibiotic-microbiota research.

Domain	Main limitations	Recommended solutions	Priority research questions
Study design	Small sample sizes; predominantly cross-sectional; lack of longitudinal follow-up; inadequate control for confounders (e.g., underlying diseases, nutrition, other medications)	Large, multicentre prospective cohort studies; longitudinal sampling from admission to discharge and beyond	How does the gut microbiota change across different phases of PICU stay, and when is the optimal time for intervention?
Microbiome analysis	Most studies use 16S rRNA sequencing, lacking functional information; non-uniform bioinformatics pipelines	Multi-omics integration (metagenomics, metabolomics, metatranscriptomics)	Which functional pathways (e.g., SCFA production, bile acid metabolism) are most predictive of clinical outcomes?
Antibiotic exposure reporting	Wide variation in metrics (DDD, DOT, days of therapy); cumulative exposure poorly quantified	Standardised reporting using multiple metrics (e.g., DOT/100pd, number of antibiotic classes, duration of anaerobic coverage)	What is the dose–response relationship between cumulative antibiotic exposure and the degree of microbiota disruption?
Intervention strategies	Timing, dose, route, and strain of probiotics not standardised; safety reporting incomplete	Rigorous RCTs with predefined safety endpoints; head-to-head comparisons of different interventions	Which interventions are effective in early (hyperinflammatory) vs. late (immunoparalysis) stages of critical illness?
Long-term outcomes	Follow-up rarely extends beyond hospital discharge; impact on growth, neurodevelopment, and allergic disease unknown	Extended follow-up (6-12 months post-discharge) with standardised assessments	Does PICU−acquired dysbiosis have lasting effects on child growth, neurodevelopment, or allergic disease?
Heterogeneity	Age, underlying disease, and baseline microbiota vary widely; one-size-fits-all approaches are unlikely to succeed	Stratified or personalised approaches based on age, disease category, and baseline microbiota (e.g., low SCFA producers vs. high Proteobacteria)	Can rapid point−of−care assays (e.g., faecal SCFA, calprotectin) guide personalised microbiota−protective therapy?
Evidence source	Frequent reliance on adult-derived and neonatal-derived data for quantitative estimates; direct applicability to PICU children uncertain	Prospective validation of key adult-derived estimates in age-stratified PICU cohorts; establish PICU-specific dose–response relationships	Do dose–response relationships from adult ICU studies (e.g., 4.01% decline in butyrate producers per 48h of anti−anaerobic antibiotics) hold in PICU children of different ages?

Evidence source and generalizability. A notable limitation is the frequent reliance on adult-derived and neonatal-derived data for several quantitative estimates. While such studies provide valuable directional hypotheses, their direct applicability to PICU children remains uncertain, reflecting a broader gap in the published literature. Similarly, the evidence base for antibiotic stewardship recommendations in the PICU remains limited; most duration-of-therapy data are derived from adult populations, and few paediatric-specific randomized trials have evaluated stewardship interventions. This limitation should be acknowledged when interpreting stewardship recommendations, and local adaptation based on available resources and patient populations is essential. We have explicitly labelled non-PICU evidence throughout the manuscript to help readers distinguish between directly applicable PICU data and evidence that requires cautious interpretation.

Confounding and comparative data. Another limitation is the absence of comparative studies directly evaluating the relative impact of antibiotics versus other modifiable factors—such as enteral nutrition, proton pump inhibitors, or opioid analgesics—on gut microbiota composition in PICU children. Consequently, the assertion that antibiotics are the most significant modifiable driver is based on the strength of antibiotic-associated evidence rather than direct head-to-head comparisons.

Intervention trial methodology. The evidence for probiotics is limited by the aggregation of different strains and species in meta-analyses, preventing strain-specific comparisons. Future RCTs should be designed to evaluate individual strains or defined multi-strain combinations with adequate power to detect strain-specific effects.

Population heterogeneity. A critical limitation across most studies is the aggregation of children from infancy to adolescence, ignoring the profound age-dependent maturation of the gut microbiota and immune system. Future studies must perform age-stratified analyses (e.g., infants [<1 y], toddlers [1–3 y], pre-pubertal [4–11 y], and adolescents [12–18 y]) to uncover differential susceptibility to antibiotic-induced dysbiosis.

Outcome assessment. The immunological consequences of antibiotic-induced dysbiosis in PICU children remain poorly characterized. Most studies have focused on microbial composition rather than functional immune endpoints, and few have examined the relationship between specific microbial changes and clinically relevant immune outcomes such as Treg differentiation, mucosal IgA production, or systemic inflammatory markers. Future research should integrate immunological phenotyping with microbiome analysis to elucidate the host–microbe interactions that mediate clinical outcomes in this vulnerable population.

Future studies should address these gaps to translate current knowledge into clinical practice. In particular, large, multicentre prospective cohorts, multi-omics integration, and personalized strategies are needed to move these concepts toward bedside application.
